# Proteomics profiling identify CAPS as a potential predictive marker of tamoxifen resistance in estrogen receptor positive breast cancer

**DOI:** 10.1186/s12014-015-9080-y

**Published:** 2015-03-21

**Authors:** Henrik J Johansson, Betzabe C Sanchez, Jenny Forshed, Olle Stål, Helena Fohlin, Rolf Lewensohn, Per Hall, Jonas Bergh, Janne Lehtiö, Barbro K Linderholm

**Affiliations:** Department Oncology-Pathology, Cancer Proteomics Mass spectrometry, Science for Life Laboratory, Karolinska Institutet, SE-171 65 Stockholm, Sweden; Department of Clinical and Experimental Medicine, Faculty of Health Sciences, Linköping University, Linköping, Sweden; Regional cancer center Southeast Sweden, County Council of Östergötland, Linköping, Sweden; Department of Oncology, Radiumhemmet, Karolinska University Hospital, Stockholm, Sweden; Department of Medical Epidemiology and Biostatistics, Karolinska Institutet, Box 281, Stockholm, 17177 Sweden; Department of Oncology, Sahlgrenska Academy and University Hospital, SE-413 45 Gothenburg, Sweden

**Keywords:** Estrogen receptor, Endocrine resistance, Receptor-positive breast cancer, Proteomics, Calcyphosine, CAPS, MX1

## Abstract

**Background:**

Despite the success of tamoxifen since its introduction, about one-third of patients with estrogen (ER) and/or progesterone receptor (PgR) - positive breast cancer (BC) do not benefit from therapy. Here, we aim to identify molecular mechanisms and protein biomarkers involved in tamoxifen resistance.

**Results:**

Using iTRAQ and Immobilized pH gradient-isoelectric focusing (IPG-IEF) mass spectrometry based proteomics we compared tumors from 12 patients with early relapses (<2 years) and 12 responsive to therapy (relapse-free > 7 years). A panel of 13 proteins (TCEAL4, AZGP1, S100A10, ALDH6A1, AHNAK, FBP1, S100A4, HSP90AB1, PDXK, GFPT1, RAB21, MX1, CAPS) from the 3101 identified proteins, potentially separate relapse from non-relapse BC patients. The proteins in the panel are involved in processes such as calcium (Ca^2+^) signaling, metabolism, epithelial mesenchymal transition (EMT), metastasis and invasion. Validation of the highest expressed proteins in the relapse group identify high tumor levels of CAPS as predictive of tamoxifen response in a patient cohort receiving tamoxifen as only adjuvant therapy.

**Conclusions:**

This data implicate CAPS in tamoxifen resistance and as a potential predictive marker.

**Electronic supplementary material:**

The online version of this article (doi:10.1186/s12014-015-9080-y) contains supplementary material, which is available to authorized users.

## Background

Consensus guidelines for adjuvant breast cancer (BC) therapy advise different treatment modalities to diminish the risk of recurrence after surgery for primary breast cancer [1]. Factors taken into account beside stage of the disease are histopathological parameters, expression of steroid receptors, overexpression or amplification of HER2, and proliferation. Adjuvant endocrine therapy for a minimum of five years postoperatively is advised to patients with BC expressing estrogen and/or progesterone receptors (ER and PgR) and half the recurrence rate in this group. However, about a third of the eligible patients will relapse during or after tamoxifen therapy and even more so patients with advanced BC [2]. A lot of effort has been made to find markers to endocrine therapy, see Musgroove and Sutherland for a review [3].

Following the work by Sørlie and colleagues that presented the intrinsic molecular subgroups of BC based on gene expression patterns, a substantial amount of information has elucidated the complexity in pathways driving the different BC subgroups. The intrinsic subgroups differ molecularly, in prognosis as well as relapse rates after different therapy modalities [4]. The two luminal subgroups originating from the well differentiated luminal layer are exclusively ER positive and have in general a favorable prognosis. However, there is a substantial heterogeneity within the ER positive groups where luminal B has been characterized by a more aggressive disease course compared to luminal A in terms of recurrence rate which could partly be explained by the proportion of HER2 positive patients in this group [4]. Genomic studies have great impact on BC classification enabling the identification of subgroups within this heterogeneous disease which is consequently taking us a step closer to personalizing therapy.

Proteomics is a mean of complementing the genomics information since mRNA and protein levels don’t always correlate [5]. Several different proteins and signaling pathways have been suggested to be part of the tamoxifen resistance mechanism, for example kinase expression levels and activity, transcription factors and their coregulators, as well as downstream intracellular events as the PIK3/AKT/mTOR pathway [3,6]. In addition, other nuclear receptors, as the retinoic acid receptor alpha are involved in tamoxifen resistance [7].

Apart from binding and inhibiting ER, tamoxifen also bind and inhibit the calcium binding protein calmodulin (CALM) [8,9]. CALM regulates many cellular protein kinases, phosphatases and transmembrane ion transporters, mainly in a calcium dependent manner. CALM interacts and modulate ER activity [10]. Another member of the EF hand motif family is Calcyphosine (CAPS) - for calcium binding and regulated by cyclic AMP through phosphorylation protein. CAPS has been suggested as an alternative calcium signaling route to CALM [11]. CAPS have high levels in endometrial tumors, whose proliferation is known to be induced by tamoxifen, compared to normal proliferative tissue [12].

Here we use mass spectrometry (MS)-based proteomics to discover potential predictive biomarkers for adjuvant tamoxifen therapy in a patient population that received adjuvant tamoxifen as the only systemic adjuvant therapy. We identified 13 proteins showing significant differential expression in relapsing patients compared to our defined control group. These were involved in processes such as calcium (Ca^2+^) signaling, metabolism, epithelial mesenchymal transition, metastasis and invasion [12-14]. Validation of calcyphosine (CAPS) in the entire clinical cohort suggests that high levels predict relapse, and that CAPS is a potential predictor of tamoxifen response.

## Results

### Experimental design and mass spectrometry based proteomic

**Figure 1:**
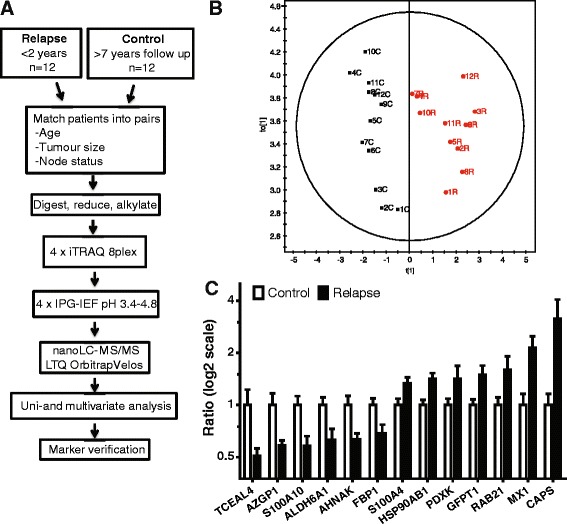
**Putative tamoxifen predictive proteins. (A)** Proteomics discovery workflow. **(B)** Score scatter plot from uni- and multivariate analysis, separating 12 matched pairs of control and relapse patients based on a 13 protein signature. Numbers indicate matched patient pairs. C (black) for control (>7 years of disease free follow up) and R (red) for relapse (within 2 years) patients. P = 2.2e-005. **(C)** Quantitative iTRAQ proteomics data showing the differences between control and relapse patients for the 13 proteins. Abbreviation: IPG-IEF, immobilized pH gradient – isoelectric focusing.

To do an unbiased search for tamoxifen predictive markers, we performed quantitative proteomics on tumor homogenates from BC patients. We selected tumors from 12 patients who relapsed within 2 years of tamoxifen treatment (referred to as relapse) and 12 patients with a disease-free follow up time of more than 7 years (referred to as control). Patients were matched into 12 pairs defined by age, tumor size, and node status. All patients were ductal and ER positive cancers (Table 1). Quantitative mass spectrometry based proteomics on these patient samples was performed by nanoLC-MS/MS using LTQ-Orbitrap Velos mass spectrometer on fractions from peptide isoelectric focusing, pH 3.4–4.8. See Figure 1A for workflow. MS based proteomics yielded a total of 3101 identified proteins, of which 550 overlapped between all the 4 iTRAQ sets and used in the analysis (Additional file 1).

**Table 1:** **Clinicopathological characteristics of patients included in the discovery proteomics**

**Feature**	**Control n =12**	**Relapse n = 12**
*Planned tamoxifen regimen*		
2 years	1	5
5 years	11	7
*Age, years*		
Median	61.6	65.1
Range	38–79	36–84
*Tumor size*		
T1	5	5
T2	4	5
T3	3	2
*Lymph-node status*		
Node-negative	5	5
Node-positive	7	7
1–3	3	2
≥4	4	5
*ER (fmol/μg DNA)*		
Median	1.8	1.0
Average	2.7	1.4
Range	0.52–9.4	0.08–4.1
*PgR (fmol/μg DNA)*		
Median	2.3	0.6
Average	4.1	2.4
Range	0–16.9	0–12.8

All patients were ER positive and received adjuvant tamoxifen as the only systemic adjuvant treatment.

### Statistical analysis of proteomics data to identify tamoxifen-predictive markers

Uni- and multivariate analysis by SAM and OPLS were used to identify potential tamoxifen predictive markers [15,16]. These statistical analyses revealed a 13 protein signature, which could separate relapse vs. control groups, P-value 2.2e-005 (Figure 1B). Principal component analysis (PCA) displayed no bias between and within the relapse and control groups (Additional file 2A-C). Protein identities and iTRAQ protein quantities of the potential 13 protein signature are shown in Figure 1C. Many of the proteins in the 13 protein panel have been connected to BC and other cancer types before (Table 2). There is connection to EMT via S100A4, an EMT marker, as well as S100A10 and AHNAK, who are protein complex components together with E-cadherin at the plasma membrane.

**Table 2:** **Connection of the 13 protein panel to cancer**

**Protein**	**Connection to cancer**	**References**
TCEAL4	Transcription elongation factor A (SII) like 4 (TCEAL4) is down-regulated in anaplastic thyroid cancer.	[34]
AZGP1	Stimulates lipid degradation. AZGP1 is a tumor suppressor in pancreatic cancer inducing mesenchymal-to-epithelial (MET) transdifferentiation by inhibiting TGF-β-mediated ERK signaling. The percentage of IHC positive stained cells in (Pancreatic Intraepithelial Neoplasia) PanIN lesions, primary and metastatic PDAC, gradually decreases from 48 to 26 and 5%, respectively.	[35]
	More malignant breast tumors showed downregulated AZGP1 mRNA.	[36]
	Low AZGP1 expression, by IHC, was associated with clinical recurrence in prostate cancer.	[37]
S100A10	S100A10 was down-regulated in breast cancer, irrespective of pathological stage.	[38]
	Complex with AnnexinA2 and AHNAK and E-cadherin at the plasma membrane.	[26,27]
	S100A10 is required for recruitment of macrophages to tumor sites and tumor growth.	[39]
ALDH6A1	Catalyzes the irreversible oxidative decarboxylation of malonate and methylmalonate semialdehydes to acetyl- and propionyl-CoA. Decreased expression with increasing grade in kidney cancer.	[40]
AHNAK	Associates with S100A10 and E-cadherin at the plasma membrane.	[26]
FBP1	Converts fructose-1,6-bisphosphate to fructose 6-phosphate in gluconeogenesis. Antagonize glycolysis. Downregulated through NF-kappaB pathway in Ras-transformed NIH3T3 cells. Restoration of FBP1 expression suppressed anchorage-independent growth.	[41]
	Loss of FBP1 by Snail-G9a-Dnmt1 complex increase glucose uptake, glycolysis and induce a cancer stem cell like characteristics.	[31]
S100A4	Involved in regulation of angiogenesis, cell survival, motility, and invasion. EMT marker.	[28,42,43]
	Staining for S100A4 is associated with poorer survival in BC.	[44]
HSP90AB1	High levels of HSP90AB1 correlates with poor prognosis in HER2-/ER+ BC.	[45]
GFPT1	Rate-limiting enzyme of hexosamine biosynthetic pathway (HBP).	[29]
	IHC analysis indicated elevated GlcNAcylation levels in breast tumor tissue as compared to adjacent tissue. GlcNAcylation was significantly enhanced in metastatic lymph nodes compared with their corresponding primary tumor tissues.	[30]
	BC cells upregulate HBP, including increased O-GlcNAcation and elevated expression of O-GlcNAc transferase (OGT), which is the enzyme catalyzing the addition of O-GlcNAc to proteins.	[46]
PDXK	Required for synthesis of pyridoxal-5-phosphate from vitamin B6. pyridoxal-5-phosphate is a prosthetic group of some enzymes.	
RAB21	Overexpression stimulates cell migration. Small GTPase Rab21 regulates cell adhesion and controls endosomal traffic of β1-integrins.	[47]
	Rab21 is required for Carcinoma-associated fibroblasts (CAFs) to promote the invasion of cancer cells. RAB21 enables the accumulation of integrin a5 at the plasma membrane and subsequent force-mediated matrix remodelling.	[48]
MX1	Stable exogenous MX1 expression inhibited in vitro motility and invasiveness of human prostate carcinoma cell line PC-3 M.	[49]
	Upregulated in a mammary carcinoma xenograft model of tamoxifen resistance.	[23]
	Upregulated in a fulvestrant-resistant cell line T47D-r on the mRNA and protein level compared to T47D.	[24]
CAPS	High expression gives bad prognosis in endometrial cancer.	[21]
	Over-expressed in ovarian cancer.	[50]
	Upregulated 30× in MCF7 ErbB2 compared to MCF7.	[51]
	Phosphorylation substrate for protein kinase A.	[11,52]

### Validation of potential predictive protein biomarkers in tumor homogenates

**Figure 2:**
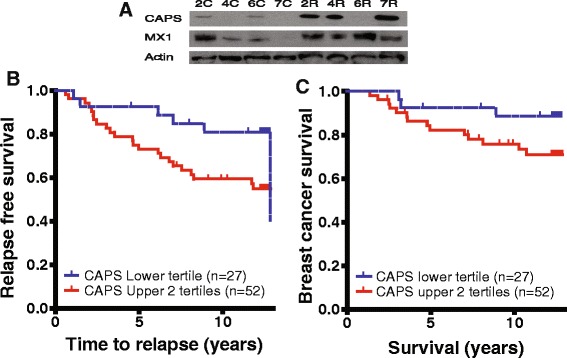
**Verification of proteomics data and identification of CAPS as a potential predictive marker for early relapse of breast cancer patients receiving tamoxifen. (A)** Western blot of 4 matched pairs of patients, randomly selected from the discovery set to verify CAPS and MX1 expression. **(B)** Relapse free survival (p = 0.049) and **(C)** Breast cancer survival (p = 0.11) of patients receiving adjuvant tamoxifen. Patients were divided by CAPS expression into lower tertile and 2 upper tertiles. See Table 2 for patient characteristics for **(B)** and **(C)**.

A positive selection marker is generally preferred over a negative marker and since our aim was to identify patients relapsing on tamoxifen, we choose to verify expression of the 2 proteins with the highest expression ratio in the relapse group, CAPS and MX1, from the proteomics data (Figure 1C). An initial verification was performed by western blot (WB) on four randomly selected matched pairs of cytosols from all 24 patients included in the study. The WB showed that both CAPS and MX1 had overall higher protein levels in the relapse group compared to control (Figure 2A). Correlation between MS and WB data were 0.8 *R*^2^ (p = 0.0037) for CAPS and 0.6 *R*^2^ (p = 0.024) for MX1 (Additional file 3).

Based on this small verification we performed protein quantification by ELISA for CAPS and MX1 on 79 and 89 breast tumor homogenates respectively. See Table 3 for clinical characteristics. Relapse-free survival (RFS) and breast cancer-specific survival (BCS) were used as primary end points, based on the time from diagnosis to the first event of loco-regional or distant recurrence, and time from diagnosis to breast cancer death, respectively.

**Table 3:** **Clinicopathological characteristics of patients included in the validation cohort**

**Feature**	**Nr (%)**	**CAPS**			
		**Q1**	**Median**	**Q4**	**P-value**
*Age, years*					
Median	63				
Range	32–87				
<50	21 (27%)	9.6	14.6	22.3	
≥50	58 (73%)	18.7	21	38.4	0.0049
*Tumor size*	45 (57%)	18.2	25.5	36.1	
T1					
T2–3	34 (43%)	11.5	20.1	31.2	0.15
*S-phase*					
<10%	50 (78%)	16.5	25.5	33	
≥10%	14 (22%)	8.5	19.7	42.9	0.29
Missing	15				
*Lymph-node status*					
Node-negative	42 (56%)	17.4	24.8	38.9	
Node positive	33 (44%)	12.6	24.3	32.0	0.33
Missing	4				
PgR (fmol/μg DNA)					
Neg (≤0.09)	23 (29%)	14.8	30.5	38.9	
Pos (>0.09)	56 (71%)	14.6	24.3	29.7	0.58

All patients were ER positive (Average ER = 4.4 fmol/μg DNA) and received adjuvant tamoxifen as the only systemic adjuvant treatment. CAPS protein levels were determined by ELISA (n = 79). Mann-Whitney’s test for significance. Abbreviation: PgR, progesterone receptor.

High protein levels of CAPS was associated with increased RFS (p = 0.049, 85% power) and BCS (p = 0.11, 80% power) (Figure 2B, C). Selection of lower tertile vs. the 2 higher tertiles for CAPS was done based on difference in HR to continuous data (Additional file 4). MX1 protein levels were not correlated to RFS or BCS in this clinical cohort (Additional file 5).

CAPS remained an independent prognostic marker in a Cox proportional multivariate analysis for RFS (HR = 3.6; p = 0.011), while nodal status (node-negative versus node-positive) (HR = 1.7; p = 0.22), tumor size (<20 mm versus ≥ 20 mm) (HR = 1.4; p = 0.43), age (HR = 1.0; p = 0.87), ER (HR = 0.88; p = 0.073) and PgR (HR = 0.96; p = 0.48) were not (Table 4). CAPS was also statistical significant factor in multivariate analysis for BCS (HR = 4.0 p = 0.043). Despite a total of 29 relapses and 17 breast cancer deaths were registered among the 79 patients with data on CAPS.

**Table 4:** **Univariate and Cox proportional multivariate analyses for recurrence (n = 29) and breast cancer death (n = 17) in the 79 patients validation cohort with ER positive breast cancer, treated with tamoxifen only as systemic adjuvant therapy**

**VARIABLES**	**RFS**				**BCS**			
	**HR**	**95% CI**		**p-value**	**HR**	**95% CI**		**p-value**
**Univariate**		**Lower**	**Upper**			**Lower**	**Upper**	
Tumor size (T1 vs. T2–3)	1.4	0.67	2.9	0.37	1.3	0.48	3.3	0.63
Node status (pos vs neg)	2.1	0.97	4.6	0.059	5.3	1.51	18.7	0.0092
CAPS (high vs low)	2.4	0.98	6.0	0.049	2.8	0.80	9.6	0.11
Age (<50 vs ≥50 years)	1.00	0.97	1.03	0.82	1.01	0.98	1.05	0.60
ER cont (fmol/μg DNA)	0.87	0.77	0.98	0.026	0.76	0.60	0.95	0.018
PgR cont (fmol/μg DNA)	0.93	0.84	1.03	0.16	0.85	0.71	1.01	0.061
CAPS (high vs low)	2.4	0.98	6.0	0.056	2.8	0.80	9.6	0.11
**Multivariate**								
Tumor size (T1 vs T2–3)	1.4	0.61	3.2	0.43	0.90	0.30	2.7	0.85
Node status (pos vs neg)	1.7	0.72	4.0	0.22	4.7	1.24	18.0	0.024
CAPS (high vs low)	3.6	1.3	9.7	0.011	4.0	1.04	15.1	0.043
Age (<50 vs ≥50 years)	1.00	0.97	1.03	0.87	1.02	0.99	1.06	0.24
ER cont (fmol/μg DNA)	0.88	0.76	1.01	0.073	0.72	0.55	0.96	0.025
PgR cont (fmol/μg DNA)	0.96	0.87	1.07	0.48	0.92	0.77	1,11	0.40
CAPS (high vs low)	3.6	1.3	9.7	0.011	4.0	1.04	15.1	0.043

*Abbreviations*: *RFS* Relapse-free survival, *BCS* breast cancer-specific survival.

Receiver operator characteristic (ROC) for CAPS yielded an area of 0.62 (p = 0.08) and 0.58 (p = 0.29) for RFS and BCS, respectively (Additional file 6).

## Discussion

The vast majority of BC patients are ER positive, making them eligible for adjuvant endocrine treatment. Tamoxifen has been the corner stone in breast cancer treatment since 40 years, although in part replaced with the aromatase inhibitors (AI) during the last decade. However, due to the marginal benefit and the severe side effects accomplished with aromatase inhibitor therapy, tamoxifen still plays an important role in breast cancer treatment. Tamoxifen alone is not sufficient in about 30% of these patients [2].

Genomics together with proteomics are on their way to decipher the complex molecular events that are responsible for the heterogeneity within subgroups of this disease [17]. In this study we compared the protein differences between patients with a relapse-free survival of more than 7 years and those exhibiting a relapse within 2 years of tamoxifen treatment. We identified a potential signature of 13 proteins that was able to significantly differentiate relapsing patients from our defined control group (Figure 1) Expression analysis of the 2 proteins with highest levels in the relapse group confirmed that high levels of CAPS, but not MX1, to be related to tamoxifen non-responsive patients.

High levels of CAPS were statistically significantly correlated to lower RFS both in uni- and multivariate analyses, including important breast cancer prognostic markers as nodal status, tumor size, age and ER and PgR levels. Several gene array based tests are validated and commercially available for breast cancer care. Some are developed to gain information about endocrine therapy. The Oncotype dx separates patients where endocrine therapy solely is enough versus patients that are in need of additional chemotherapy [18]. The majority of markers included in these profiles have been linked to proliferation. Endopredict can identify ER+, HER2 negative patients with an increased likelihood of development of late distant metastasis [19]. Interestingly, Endopredict include CALM2, a Ca^2+^ binding protein. Our ROC analysis also indicates that CAPS protein levels needs to be used together with other markers to obtain good sensitivity and specificity to predict outcome. However, we can not exclude CAPS as a prognostic factor with the clinical cohort used in this study.

CAPS is a Ca^2+^ binding protein whose synthesis and activation is induced by the cAMP cascade (PKA) and connected to signaling for cellular proliferation and differentiation [11]. Interestingly, tamoxifen also has effects on Ca^2+^ signaling, aside from its antiestrogenic properties by binding to and inhibiting CALM activity [20]. Our data of CAPS overexpression in non-responsive patients raises the hypothesis that tamoxifen fails to inhibit the calcium signaling response in the cell, and that high CAPS expression can function as an alternative compensatory mechanism when CALM is inhibited. CAPS has previously been suggested to be an alternative signaling step to CALM [11]. Moreover, recent studies found higher CAPS levels in endometrial cancer compared to normal proliferative tissue [12,21]. It is of note that tamoxifen has been shown to increase the risk for endometrial cancer due to proliferative effects of the drug in this tissue [22].

The MX1 results are surprising since MX1 showed increased mRNA and protein levels in tamoxifen and fulvestrant resistance models [23,24]. The number of psms for quantification differs between the 4 iTRAQ sets, with 1, 3, 8, and 27 psms used for iTRAQ quantification, which could have introduced quantification bias. However, the negative result for MX1 with ELISA is probably due to antibody effects, *i.e.* different or mixed epitope recognition by the antibody compared to proteomics data. In comparison, CAPS had 28, 5, 21 and 14 psms used for quantification in the 4 different iTRAQ 8plex sets. Hence, CAPS had a more robust MS quantification that is less sensitive to noise and more likely to identify the major protein variant that will ease ELISA validation.

Previous studies using DNA microarrays and proteomics have shown upregulation of MX1 in vivo and in vitro in cells resistant to tamoxifen (*in vivo*) and to the ER downregulator fulvestrant (*in vitro*) [23,24]. A recent in vitro study implicated the proliferative PIK3/AKT pathway as part of the regulatory cascade inducing MX1 expression in response to IFNα [25]. The upregulation of MX1 in the relapsing patients may be a consequence of induction of growth signaling through various pathways.

Some draw backs with the present study must be discussed. So far, proteomics have been very labor intensive making discovery work in a large number of tumors difficult. The small sample set for the discovery phase potentially increase putative markers without any value. Larger test sets of at least 100 patients in each group are probably needed for identification of more robust markers. Another drawback is that lobular patients were excluded. These patients are often ER positive and subjected to adjuvant endocrine treatment, thus the role of CAPS has to be studied in all ER positive breast cancer types. However, an advantage of proteomics is the direct identification of a particular protein, or a set of proteins, which are measurable by more time-effective, simpler and less costly routine analysis methods such as IHC and ELISA in the clinic.

The potential 13 protein panel shows connection to breast and other forms and cancer (Table 2). Among the proteins, there is connection to invasion, motility and epithelial mesenchymal transition (EMT) by RAB21, MX1, S100A4, S100A10, AHNAK, AZGP1. S100A10, AHNAK and E-cadherin form protein complexes on the plasma membrane [26,27]. S100A4 (also known as FSP1 and MTS-1) is an EMT marker [28]. There is also a connection to metabolic changes by GFPT1, PDXK, FBP1, ALDH6A1 and AZGP1. GFPT1 is the rate limiting enzyme into the hexosamine synthesis pathway and increased downstream GlcNAcylation is increased in breast tissue compared to normal tissue [29,30]. PDXK catalyze the synthesis of the prosthetic group pyridoxal-5-phosphate from vitamin B6. The Snail-G9a-DNMT1 protein complex has been shown not only to decrease E-cadherin levels but also FBP1, increasing glucose uptake, inducing glycolysis and cancer stem cell like characteristics [31].

## Conclusions

We have performed MS based proteomics on 12 tumors from relapse and 12 tumors from control patients and associated a 13 protein panel with tamoxifen resistance. Validation of the highest expressed protein in the relapse group, CAPS, in the whole cohort implicate CAPS as a potential predictive factor in ER positive breast cancer receiving adjuvant tamoxifen.

## Methods

### Patient characteristics and selection

The study investigate differences in protein expression profile between patients exhibiting relapses within two years of tamoxifen therapy (referred to as relapse group) and patients with a relapse-free survival of more than seven years (referred to as control group) included twenty-four patients (12 patients in each group). The reason for choosing a relapse-free period of more than 7 years was to include the possible hang-over effect by tamoxifen. In order to avoid bias from biological differences, the two groups were matched for tumor size, nodal status, and age. Only ductal cancers were included. Data on histopathological grade was not available. These patients originate from a BC material previously described in detail [32]. In short, a total of 402 patients with ER positive BC subjected to adjuvant tamoxifen as the only adjuvant systemic therapy (no chemotherapy allowed) were identified from a larger consecutive cohort of 711 patients diagnosed with a primary breast stage I-III from 1991 to 1996. Patients with a locally advanced BC, displaying distant metastases at diagnosis, or having received neoadjuvant therapy, were excluded from the cohort of 402 patients. The median age of patients included in the present study was 61.6 years for the control group and 65.1 for the relapse group. A description of these patients’ characteristics is shown in Table 1. Being unable to retrieve S-phase data for all 24 patients, this factor was excluded. The study was approved by the research ethical boards of Linköping University Hospital, Linköping, Sweden. During the study period, there was no requirement to receive an informed consent from each patient for storage and usage of the remaining tumor homogenates according to Swedish law.

### Preparation of tumor homogenates

Representative tumor tissue was homogenized in a microdismembrator (Braun, Melsungen, Germany) and suspended in cold potassium phosphate buffer (5 mM, pH 7.4, 10% glycerol v/v, 1 mM dithiothreitol). Supernatants containing the cytosolic fractions were collected after refrigerated centrifugation at 20,000 g, used for steroid receptor content analysis and stored at –70°C. The pellet fractions were analyzed by the method of Burton, in order to evaluate DNA concentration.

### Sample preparation for mass spectrometry

Tumor homogenates (cytosols) were acetone precipitated and pellets were dissolved in 1% SDS, which were diluted to 0.1% before protein concentration determination with Bio-rad DC protein assay. Triethylammoniumbicarbonat was added to each sample to give a final concentration of 0.5 M. Proteins were reduced by adding tris-(2-carboxyethyl) phosphine (TCEP) and alkylated by methyl methanethiosulfonate (MMTS). Trypsin (modified sequence grade, Promega, Madision WI, USA) was added (1:20, trypsin:protein) and the samples were incubated overnight at 37°C. iTRAQ labelling of the peptides were done according to the manufacturer’s protocol (Applied Biosystems) and cleaned by a strata-X-C-cartridge (Phenomenex). Four different iTRAQ 8plex sets was used to include all patients, and the 4 sets were connected by 2 iTRAQ channels with pooled samples, one for the 12 controls (PC) and one for the 12 relapse (PR) samples. Three pairs of matched control (115, 116, 117) and relapse (118, 119, 121) samples were included together with the pooled samples (113, 114) in each iTRAQ 8plex set.

### Peptide separation

The iTRAQ labelled peptides, 360 μg for each of the 4 iTRAQ sets, were separated by immobilized pH gradient - isoelectric focusing (IPG-IEF) on a narrow range pH 3.4–4.8 strip as described by Branca *et al.* [33]. Peptides were extracted from the strips by a prototype liquid handling robot, kindly supplied by GE Healthcare Bio-Sciences AB. A plastic device with 72 wells was put onto each strip and 50 μl of MQ was added to each well. After 30 minutes incubation, the liquid was transferred to a 96 well plate and the extraction was repeated 2 more times. The extracted peptides were dried in speedvac and dissolved in 3% acetronitrile (ACN), 0.1% formic acid.

### NanoLC-MS/MS analysis

Before analysis on the LTQ-Orbitrap Velos (Thermo Fischer Scientific, San Jose, CA, USA), peptides were separated using an Agilent 1200 nano-LC system. Samples were trapped on a Zorbax 300SB-C18, and separated on a NTCC-360/100-5-153 (Nikkyo Technos., Ltd) column using a gradient of A (3% ACN, 0.1% FA) and B (95% ACN, 0.1% FA), ranging from 3% to 40% B in 45 min with a flow of 0.4 μl/min. The LTQ-Orbitrap Velos was operated in a data-dependent manner, selecting 5 precursors for sequential fragmentation by CID and HCD, and analyzed by the linear iontrap and orbitrap, respectively. The survey scan was performed in the Orbitrap at 30.000 resolution (profile mode) from 300–2000 m/z, using lock mass at m/z 445.120025, with a max injection time of 500 ms and AGC set to 1 × 10^6^ ions. For generation of HCD fragmentation spectra, a max ion injection time of 500 ms and AGC of 2 × 10^5^ were used before fragmentation at 50% normalized collision energy. For FTMS MS2 spectra, normal mass range was used, centroiding the data at 7500 resolution. Peptides for CID were accumulated for a max ion injection time of 200 ms and AGC of 3 × 10^4^, fragmented with 35% collision energy, wideband activation on, activation q 0.25, activation time 10 ms before analysis at normal scan rate and mass range in the linear iontrap. Precursors were isolated with a width of 2 m/z and put on the exclusion list for 60 s. Single and unassigned charge states were rejected from precursor selection.

### Peptide and protein identification

All Orbitrap data was searched by Mascot 2.2 (Matrix Science Limited, London, UK) under the software platform Proteome Discoverer 1.1 (Thermo) against the human swissprot database (build 57.13) and results were limited to a false discovery rate of <1%. Precursor mass tolerance was set to 10 ppm, and product mass tolerances of 0.015 Da for HCD-FTMS and 0.7 Da for CID-ITMS were used. Oxidized methionine was set as dynamic modification and methylthio, N-terminal 8plex iTRAQ, and lysyl 8plex iTRAQ as fixed modifications. Quantification of iTRAQ 8plex reporter ions was done by Proteome Discoverer on HCD-FTMS tandem mass spectra using an integration window tolerance of 20 ppm. Proteins with 1 or more of 1% FDR confident peptides were used in the following data analysis.

### Identification of potential biomarkers

Potential biomarkers were selected by multivariate analysis of the BC patient samples with orthogonal partial least square (OPLS) analysis using Simca software, using the S-plot and loading plot described by Wiklund *et al.* 2008 and univariate analysis corrected for multiple testing using SAM [15,16].

### Western blot and ELISA

The randomly selected 8 patients included in the verification represent 4 control and 4 relapse patients from all 24 patients included in the study. 30 μg of tumor homogenate per sample were run on SDS-PAGE gels (NuPage Bis Tris 4–12%, Invitrogen), blotted and subsequently incubated overnight with CAPS (sc-134298), (Santa Cruz Biotech) and MX1 (HPA030917) (Sigma-Aldrich). After incubation with anti-rabbit or anti-mouse IgG-HRP Conjugate (BioRad), bands were detected using ECL™ Western Blotting Detection Reagents (Amersham). Commercially available enzyme-linked immune sorbent assays (ELISA) for quantification of CAPS (E92359Hu) and MX1 (E80763Hu) were performed according to the protocols of the manufacturer (USCN, Cologne, Germany).

### Statistical analysis of validation cohort

Differences in CAPS levels in relation to patient’s age and tumor characteristics were analyzed with Mann-Whitney’s test. Relapse-free survival (RFS) and breast cancer-specific survival (BCS) were chosen as primary end points, based on the time from diagnosis to the first event of loco-regional or distant recurrence, and time from diagnosis to breast cancer death, respectively. Survival curves and probabilities of RFS and BCS were estimated using the Kaplan-Meier method. Hazard ratios were calculated using Cox hazard regression analysis. Multivariate Cox models included the variables age, nodal status, tumor size, ER and PgR levels and CAPS levels. The software STATISTICA 10 (StatSoft Inc., Tulsa, OK, USA) was used for statistical calculations. P-values less than 0.05 in two-sided tests were considered significant. Graphpad prism 6 was used to visualize survival curves and perform ROC and correlation analysis. Power calculations were done using Graphpad StateMate 2.00.

## Additional files

Additional file 1:
**Proteomics data.** Protein identifications in the discovery cohort. Additional file 2:
**Principal component analysis (PCA) of quantitative data from overlapping protein identifications between the iTRAQ sets.** (A) Both control and relapse patients, denoted C and R, respectively. (B) Control patients (C) Relapse patients. Additional file 3:
**Correlation between MS and WB data.** Correlation between MS and WB data for (A) CAPS and (B) MX1. Additional file 4:
**Continuous vs. tertile analysis of CAPS.** Table showing continuous vs. tertile analysis of CAPS ELISA data in validation cohort. Additional file 5:
**Survial analysis for MX1.** Relapse free survival (A) and breast cancer survival (B) for MX1. MX1 was not associated with relapse free or overall survival. Additional file 6:
**ROC analysis for CAPS.** ROC analysis using CAPS measurements by ELISA for (A) relapse and (B) overall survival.
