# Supplementary figures and images for: Proteomics profiling identify CAPS as a potential predictive marker of tamoxifen resistance in estrogen receptor positive breast cancer
Source: Clin Proteomics. 2015 Mar 21;12(1):8. doi: 10.1186/s12014-015-9080-y (PMC4389343; doi:10.1186/s12014-015-9080-y)

A

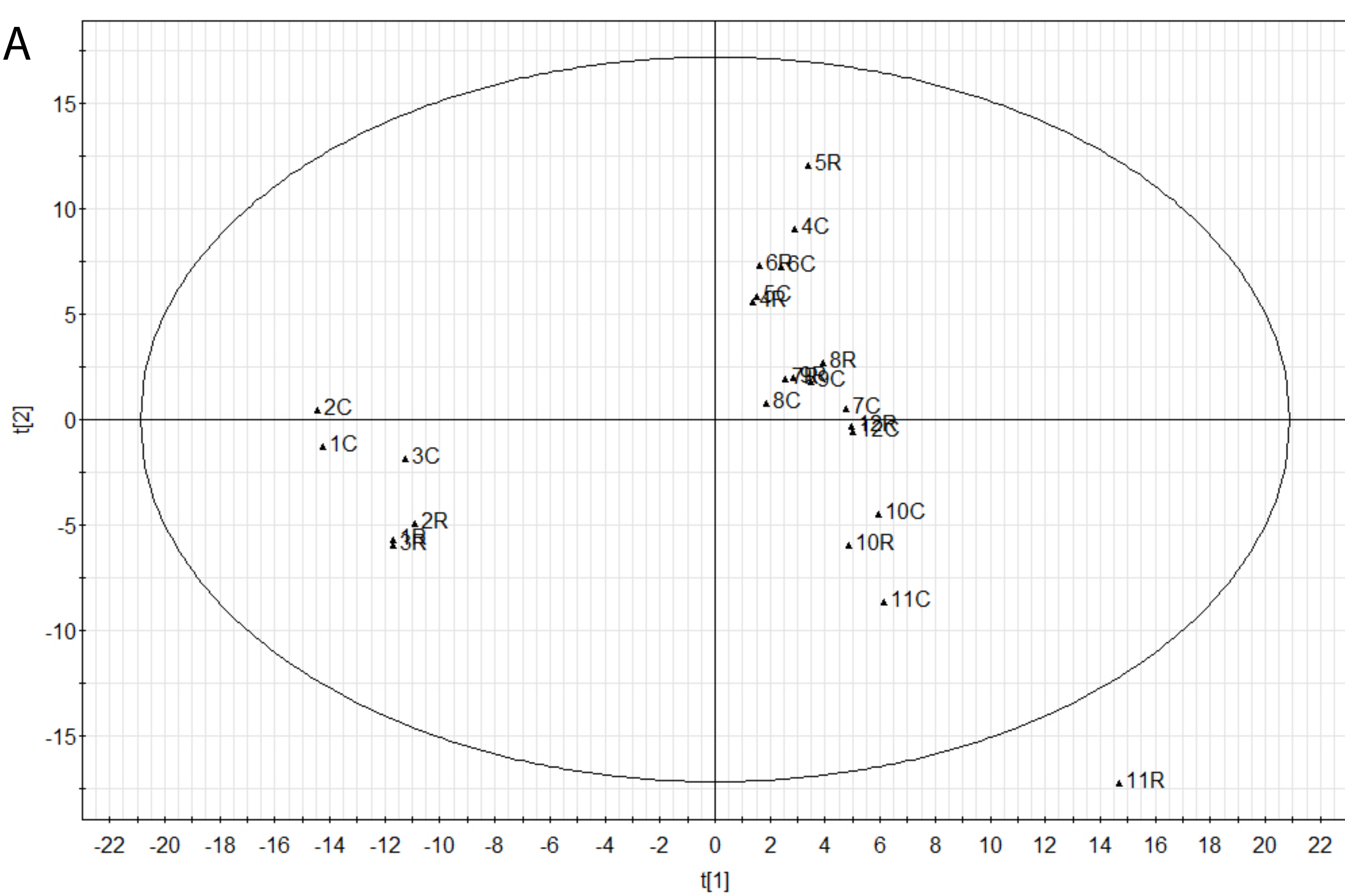

B

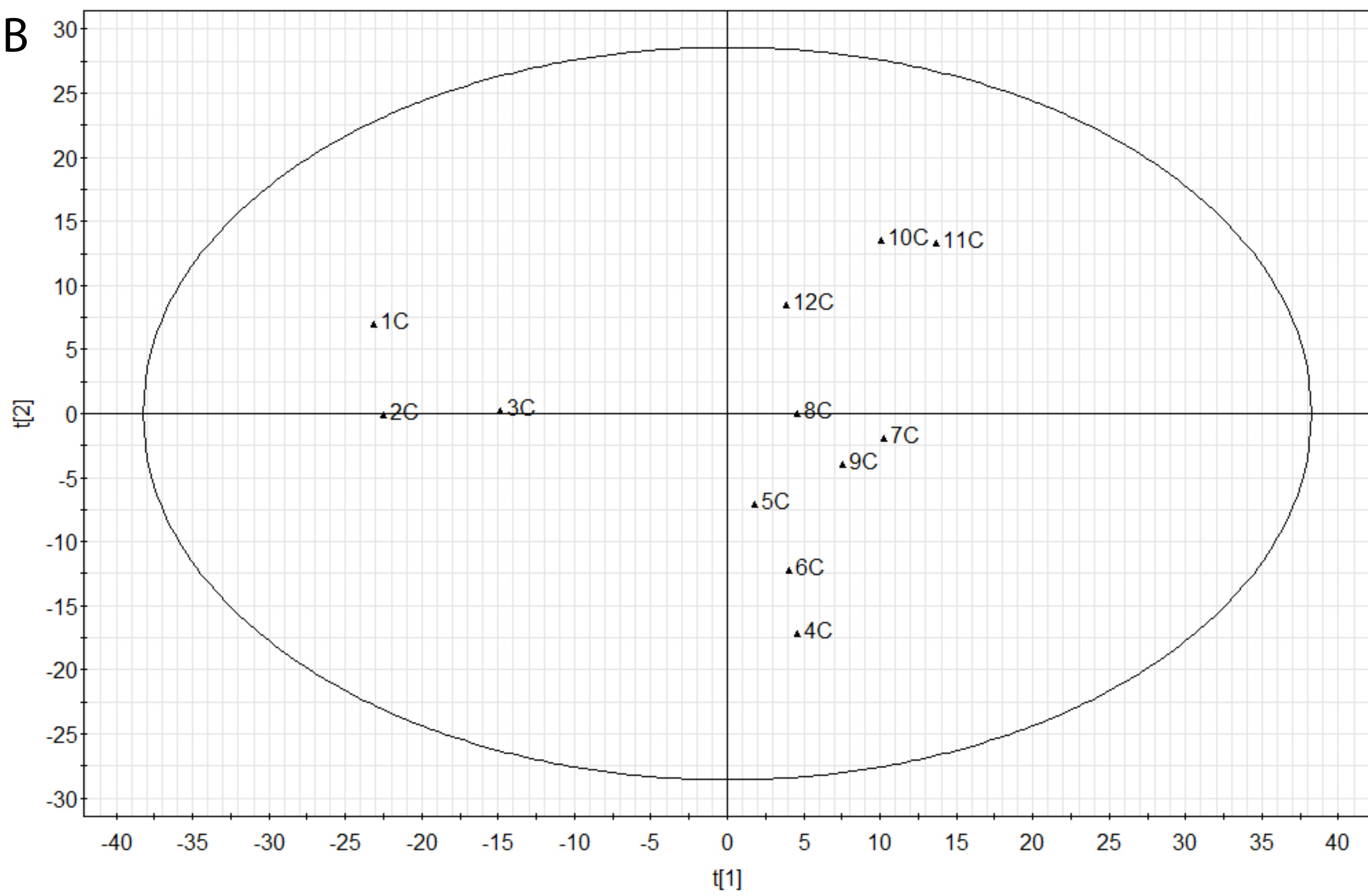

C

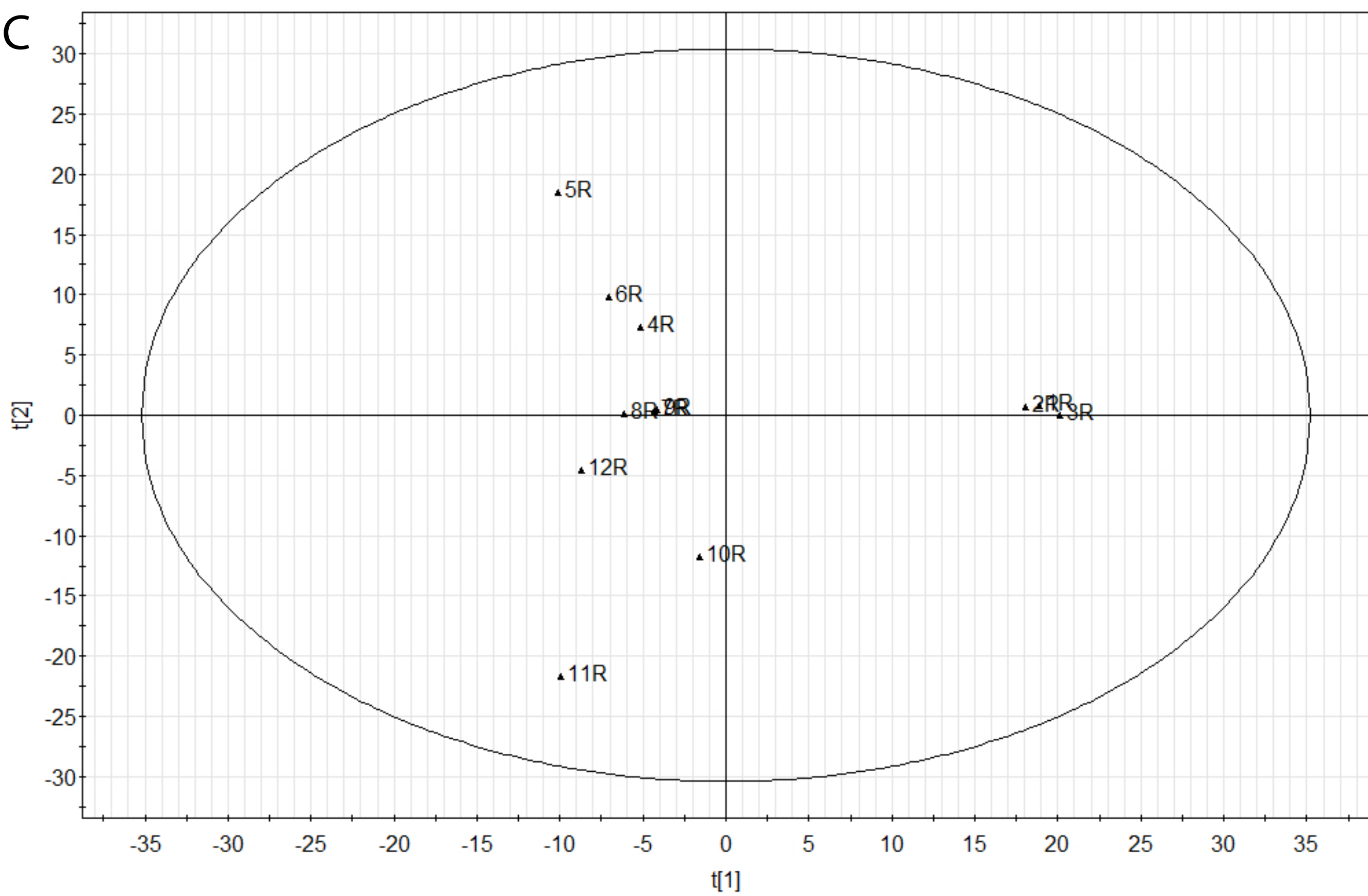

Supplement: Additional file 2: — Principal component analysis (PCA) of quantitative data from overlapping protein identifications between the iTRAQ sets. (A) Both control and relapse patients, denoted C and R, respectively. (B) Control patients (C) Relapse patients. [file 12014_2015_9080_MOESM2_ESM.pdf]

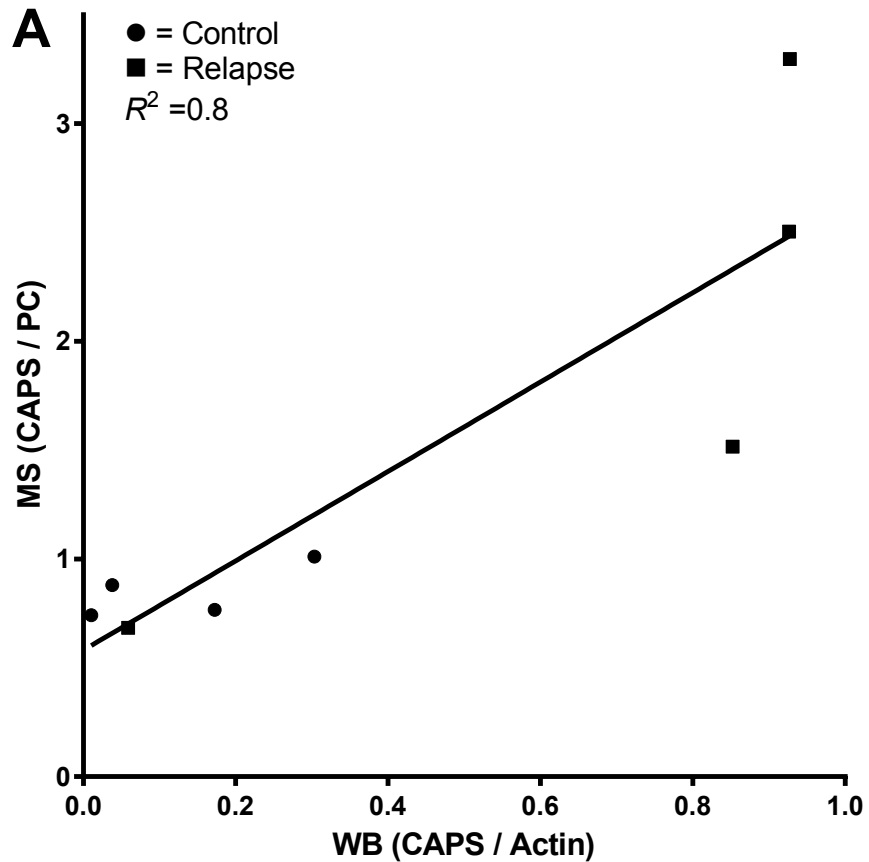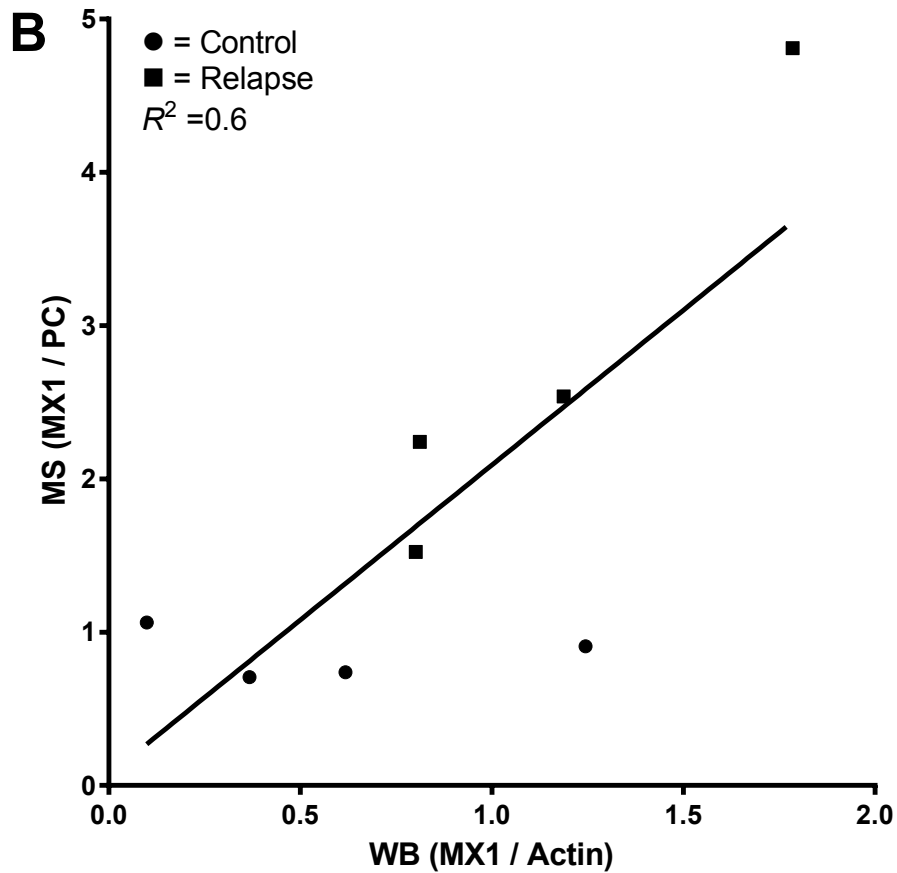

Supplement: Additional file 3: — Correlation between MS and WB data. Correlation between MS and WB data for (A) CAPS and (B) MX1. [file 12014_2015_9080_MOESM3_ESM.pdf]

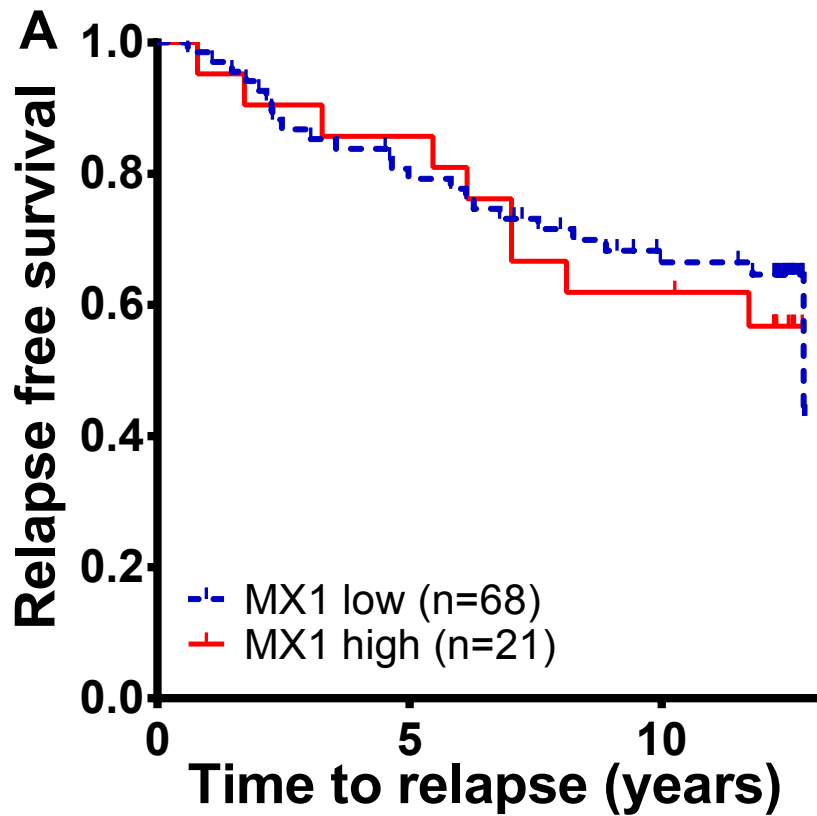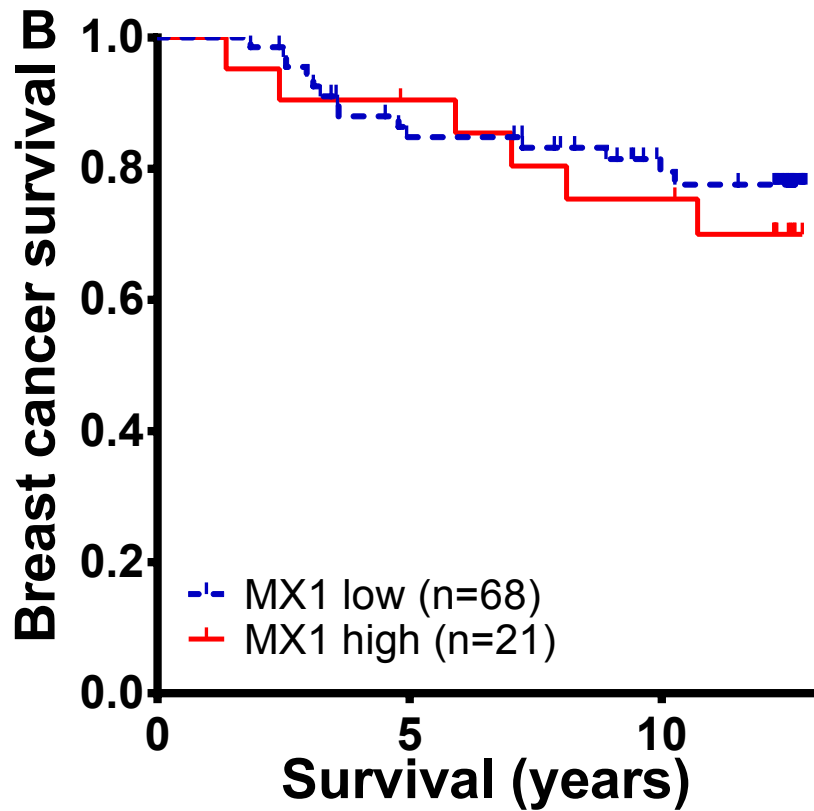

Supplement: Additional file 5: — Survial analysis for MX1. Relapse free survival (A) and breast cancer survival (B) for MX1. MX1 was not associated with relapse free or overall survival. [file 12014_2015_9080_MOESM5_ESM.pdf]

**A****Relapse**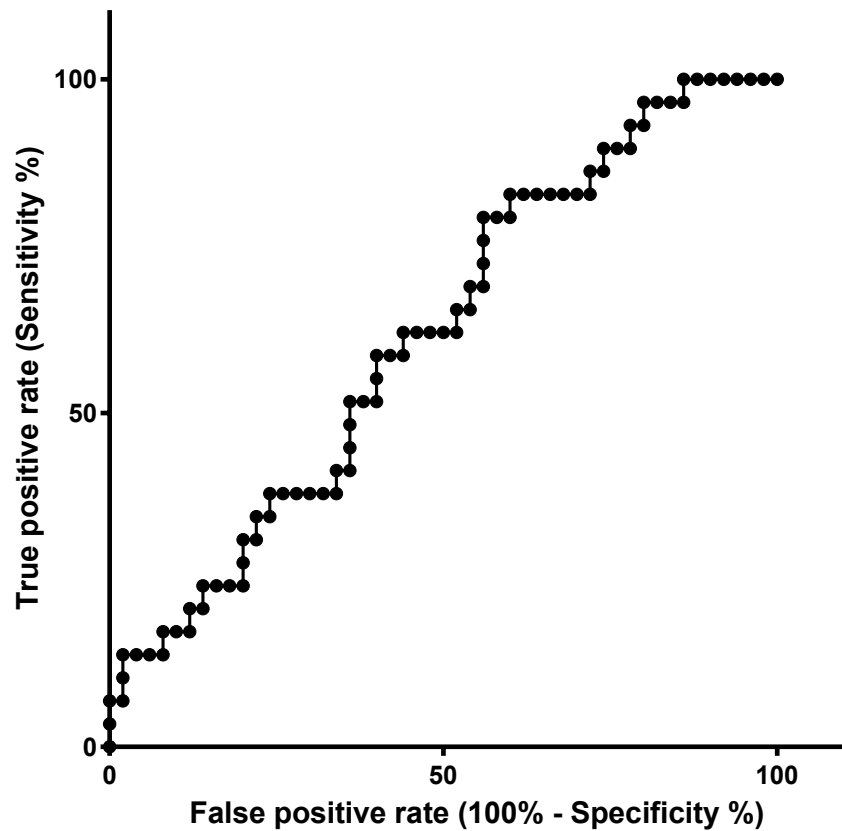**B****Survival**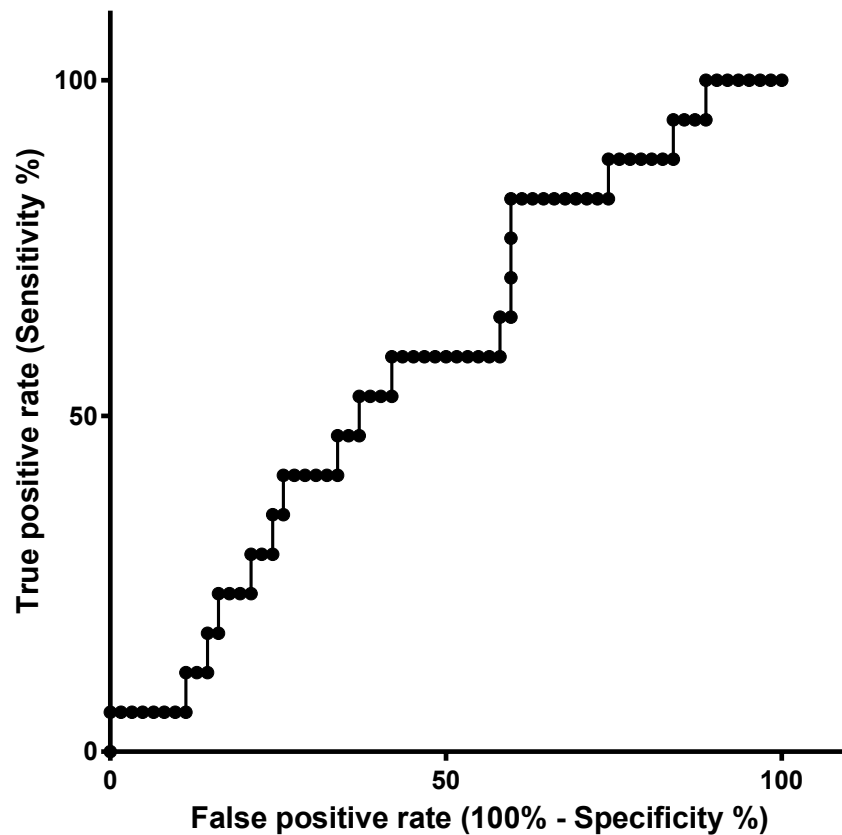

Supplement: Additional file 6: — ROC analysis for CAPS. ROC analysis using CAPS measurements by ELISA for (A) relapse and (B) overall survival. [file 12014_2015_9080_MOESM6_ESM.pdf]
