# Supplementary material for: Proteomics profiling identify CAPS as a potential predictive marker of tamoxifen resistance in estrogen receptor positive breast cancer
Source: Clin Proteomics. 2015 Mar 21;12(1):8. doi: 10.1186/s12014-015-9080-y (PMC4389343; doi:10.1186/s12014-015-9080-y)
Supplement: Additional file 4: — Continuous vs. tertile analysis of CAPS. Table showing continuous vs. tertile analysis of CAPS ELISA data in validation cohort. [file 12014_2015_9080_MOESM4_ESM.docx]

|  |  | **Recurrence** | |  |  |  | **Breast cancer death** | |  |
| --- | --- | --- | --- | --- | --- | --- | --- | --- | --- |
|  | HR | 95% CI | | p-value |  | HR | 95% CI | | p-value |
|  |  | Lower | Upper |  |  |  | Lower | Upper |  |
| CAPS continuous | 1.02 | 1.00 | 1.04 | 0.085 |  | 1.01 | 0.98 | 1.05 | 0.33 |
| CAPS tertiles |  |  |  |  |  |  |  |  |  |
| t1 | 1.0 |  |  |  |  |  |  |  |  |
| t2 | 3.0 | 1.07 | 8.2 | 0.036 |  | 2.7 | 0.70 | 10.5 | 0.15 |
| t3 | 2.4 | 0.89 | 6.7 | 0.084 |  | 2.8 | 0.73 | 11.0 | 0.13 |
